# Sex Disparities in the Clinical Characteristics, Synchronous Distant Metastasis Occurrence and Prognosis: A Pan-cancer Analysis

**DOI:** 10.7150/jca.50536

**Published:** 2021-01-01

**Authors:** Yutong Wang, Ziqian Zeng, Mingshuang Tang, Min Zhang, Ye Bai, Huijie Cui, Yao Xu, Xu Guo, Wenjuan Ma, Guijun Xu, Lisha Qi, Jingyi Wang, Siyu Chen, Dongqing Gu, Min Mao, Xin Wang, Chao Zhang

**Affiliations:** 1 Department of Health Management Center (Epidemiology and Biostatistics), First Affiliated Hospital, Army Medical University, Chongqing 400038, China.; 2 Department of Epidemiology, Chengdu Medical College, Chengdu, 610599, Sichuan province, China.; 3 Department of Epidemiology and Health Statistics, School of Public Health and Management, Chongqing Medical University, Chongqing 400038, China.; 4 Department of Bone and Soft Tissue Tumours, Tianjin Medical University Cancer Institute and Hospital, National Clinical Research Center for Cancer, Key Laboratory of Cancer Prevention and Therapy, Tianjin's Clinical Research Center for Cancer, Tianjin 300060, China.; 5 Department of Orthopedics, Cangzhou Central Hospital, Cangzhou, Hebei, 061000, China.; 6 Department of Breast Imaging, Tianjin Medical University Cancer Institute and Hospital, National Clinical Research Center for Cancer, Key Laboratory of Cancer Prevention and Therapy, Tianjin's Clinical Research Center for Cancer, Tianjin 300060, China.; 7 Department of Orthopedics, Tianjin Hospital, Tianjin 300060, China.; 8 Department of Pathology, Tianjin Medical University Cancer Institute and Hospital, National Clinical Research Center for Cancer, Key Laboratory of Cancer Prevention and Therapy, Tianjin's Clinical Research Center for Cancer, Tianjin, China.; 9 Department of Pathology and Southwest Cancer Center, First Affiliated Hospital, Army Medical University, Chongqing 400038, China.

**Keywords:** SEER, sex disparities, distant metastasis, prevalence, prognosis

## Abstract

**Background:** This study aims to assess the sex disparities in clinical characteristics and synchronous distant metastasis occurrence at diagnosis, as well as the subsequent prognosis in non-sex-specific cancers.

**Methods:** The study included details from patients diagnosed with non-sex-specific cancers, during the period from 2010 to 2016, in the Surveillance, Epidemiology, and End Results (SEER) program. The distant metastasis prevalence and subsequent survival time were summarized in the total population and the population with specific cancers of different systems. The multivariable logistic and the Cox proportional hazards regressions were applied to evaluate the sex effect on distant metastasis occurrence and prognosis. The results were combined using meta-analysis.

**Results:** Across all non-sex-specific cancers, the pooled prevalence of distant metastasis was 15.2% (95% CI: 14.7-15.7%) and 7.1% (95% CI: 6.8-7.3%) for males and females, respectively. The pooled median survival time was 8.40 months (95% CI: 7.99-8.81) for male patients and 9.40 months (95% CI: 8.84-10.02) for female patients. After combining all non-sex-specific cancers, male patients displayed a higher distant metastasis occurrence than females (pooled OR=1.06, 95% CI: 1.04-1.08; *P<*0.01), as well as worse overall survival after distant metastasis (pooled HR=1.08, 95% CI: 1.05-1.10; *P*<0.01). The sex differences were more significant in patients younger than 65 years (*P<*0.01). Additionally, the sex influence on prognosis was most predominant amongst patients from Asian or Pacific Islander ethnic groups.

**Conclusion:** Male gender appears to be an independent risk factor associated with the occurrence and prognosis of synchronous distant metastasis. Therefore, sex-specific preventions and treatments should become the focus of future research.

## Introduction

It is well established that gender plays an important role in the etiology, diagnosis, and prognosis of cancer 1-3. These gender effects might be attributed to differences in environmental, biological, and behavioral factors between the sexes, including exposure to carcinogens, hormonal axis, comorbidities, tumor biology, molecular variations, health care utilization, and response to therapies 2,4-6. Higher risks for cancer incidence and mortality were observed in males for a vast majority of sites at most ages 7-10. These findings resulted in the hypothesis that gender might affect different stages of cancer progression 11-13. Recent studies suggested that females were protected against metastasis occurrence in melanoma, esophageal cancer, pancreatic cancer, and thyroid carcinoma 14-17. Moreover, higher overall survival and cancer-specific survival were observed in females with metastatic lung and gastric cancer than in male patients 18,19. Furthermore, gender-related signatures were exhibited in up to 53% of clinically actionable genes, which indicated the necessity of sex-specific treatment for therapeutic targets with a strong gender effect 1.

Despite the significant progress that has been made in early detection and tumor growth inhibition, limited improvement has been achieved using preventive and therapeutic regimens for metastatic cancer 20,21. Around 67-90% of cancer-related deaths were attributed to the metastasis of tumor cells rather than to the primary tumors 22,23. When distant metastasis was detected, patients confronted a decrease of approximately 31-81% in the five-year relative survival rate, compared to those with localized carcinoma 24. However, there have been few studies based on a comprehensive data source with a large sample size that have systematically investigated gender disparities in metastatic risk and prognosis across all cancers. Identifying the discrepancies in metastatic characteristics and prognosis is essential to establish a more profound understanding of cancer etiology and pathogenesis 25. Patients can thus benefit from sex-specific metastatic screening, prevention, and treatment 25,26.

Based on data extracted from the Surveillance Epidemiology, and End Results (SEER) database, this study aimed to assess the sex disparities in the clinical characteristics and synchronous distant metastasis occurrence at diagnosis and to evaluate the sex disparities in prognosis across all cancers.

## Materials and Methods

### Data source and cohort selection

Data were obtained from the National Cancer Institute (NCI) SEER program, which comprised of detailed records of cancer incidence and survival for approximately 34.6% of the U.S. population. For the present study, patients were selected when diagnosed with malignant cancers, as confirmed by International Classification of Diseases for Oncology, 3rd edition (ICD-O-3) codes, from the SEER cohort within the period between 2010 and 2016. Within this cohort, patients were excluded if the diagnosis was achieved only after autopsy or via death certificate, or without clear distant metastasis information, or diagnosed with breast or genital system cancers (e.g., ovarian, cervical, and prostate), and with cancer types with <100 sample size. Therefore, the final study cohort comprised of 1,180,368 patients, meeting the research required criteria.

### Statistical analysis

The patients' demographic and clinical characteristics were described by number and percentage (N, %). The categorical variables were compared using the Pearson chi-square test, while the ordinal categorical variables were compared using the rank-sum test. Patients were stratified into two groups according to median age (<65 years vs. ≥65 years). Within male and female patients with different cancer sites, the prevalence of distant metastasis was calculated as the metastatic percentage of the total number of cancer patients. Multivariable logistic regression was applied to determine the association of gender on the occurrence of distant metastasis in non-sex-specific cancers. Demographic and clinical characteristics (age, race, marital status, income, insurance, differentiated grade, T stage, and N stage) were included in the regression model for adjustments. Meta-analysis was applied to summarise the pooled prevalence of distant metastasis, as well as the adjusted male-to-female odds ratios for the total population and cancers of different systems.

Additionally, the Kaplan-Meier analysis was utilized to estimate median survival time (M±SE) and to compare the survival time of patients diagnosed with distant metastasis in both sex groups. Multivariable Cox proportional hazards regression model was conducted to evaluate the effect of male sex on the overall survival of cancer patients with distant metastasis. The model adjusted a series of variables at diagnosis, including age, race, marital status, income, insurance, differentiated tumor grade, T stage, N stage, number of metastatic sites, surgery on the primary site, receiving radiation therapy, and receiving chemotherapy. Meta-analysis was also applied to combine the survival time of patients with distant metastasis and summarise the adjusted effects of male sex on the survival of patients with metastatic cancers of different systems and the overall survival of all cancer patients with distant metastasis. Moreover, age (<65 years vs. ≥65 years) and race (White vs. Black vs. American Indian or Alaska native vs. Asian or Pacific Islander) stratified analyses were undertaken to investigate the effects of male sex on the distant metastasis occurrence and the prognosis.

Data were obtained from the SEER program, using SEER*Stat Software version 8.3.5. SPSS 23.0 (SPSS Inc., Chicago, IL, USA) was utilized for statistical analyses. Meta-analysis and forest plots were generated with the Comprehensive Meta-analysis version 3.3 (Biostat, Englewood, NJ, USA). All statistical tests were two-sided and significant levels were set at *P* <0.05.

## Results

### Population demographic and clinical characteristics

**Figure 1:**
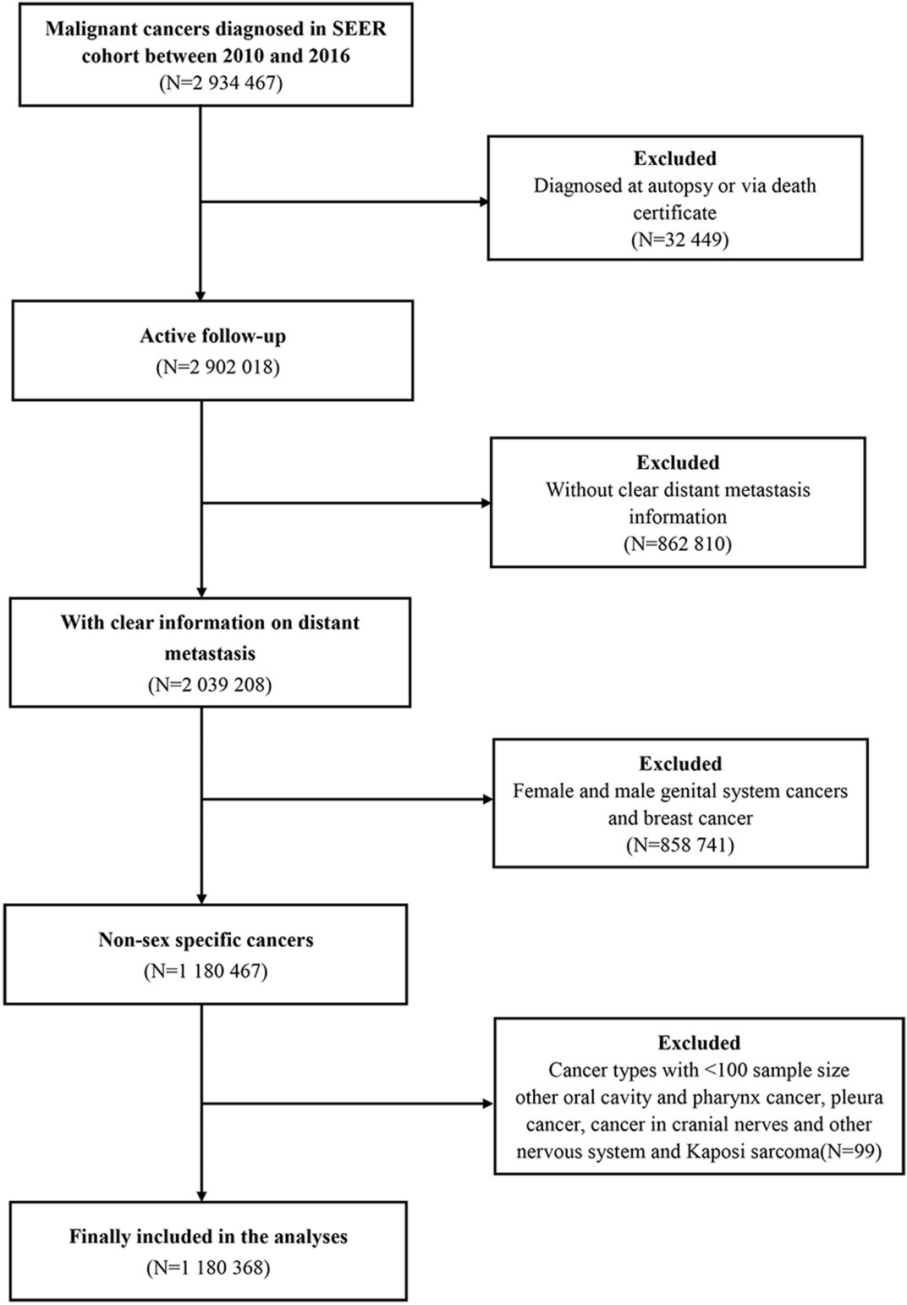
Flow-chart of the non-sex-specific cancer patient selection procedure.

The patient selection procedure is illustrated as a flowchart, shown in **Figure 1.** Once included in the study, the patients' demographic distribution and clinical characteristics are displayed in **Table 1.** A total of 1,180,368 eligible patients were selected in the statistical analysis. The rates of male and female patients accounted for 56.0% and 44.0%, respectively. The median age at diagnosis was 65.00±14.82 years for the entire patients' cohort. On the other hand, the median age at diagnosis was 65.00±13.58 years and 65.00±16.24 years for male and female patients, respectively. A total of 279,573 (23.7%) patients were detected with distant metastasis at diagnosis [N=157450, (23.8%) for male and N=122123, (23.5%) for female]. In general, male patients were more likely to be in a married relationship and be covered by medical insurance and had lower income levels than female patients (*P*<0.01).

Regarding clinical characteristics, male patients suffered from higher tumor differentiated grade, advanced tumor, and nodal stage. They appeared to be predisposed to develop distant metastasis, whether in the liver, lung, or bone (*P*<0.01). Additionally, compared with female patients, male patients were more likely to receive radiation therapy and chemotherapy, and they were less likely to undergo surgical treatment (*P*<0.01).

### Sex disparities in the distant metastasis prevalence

**Figure 2:**
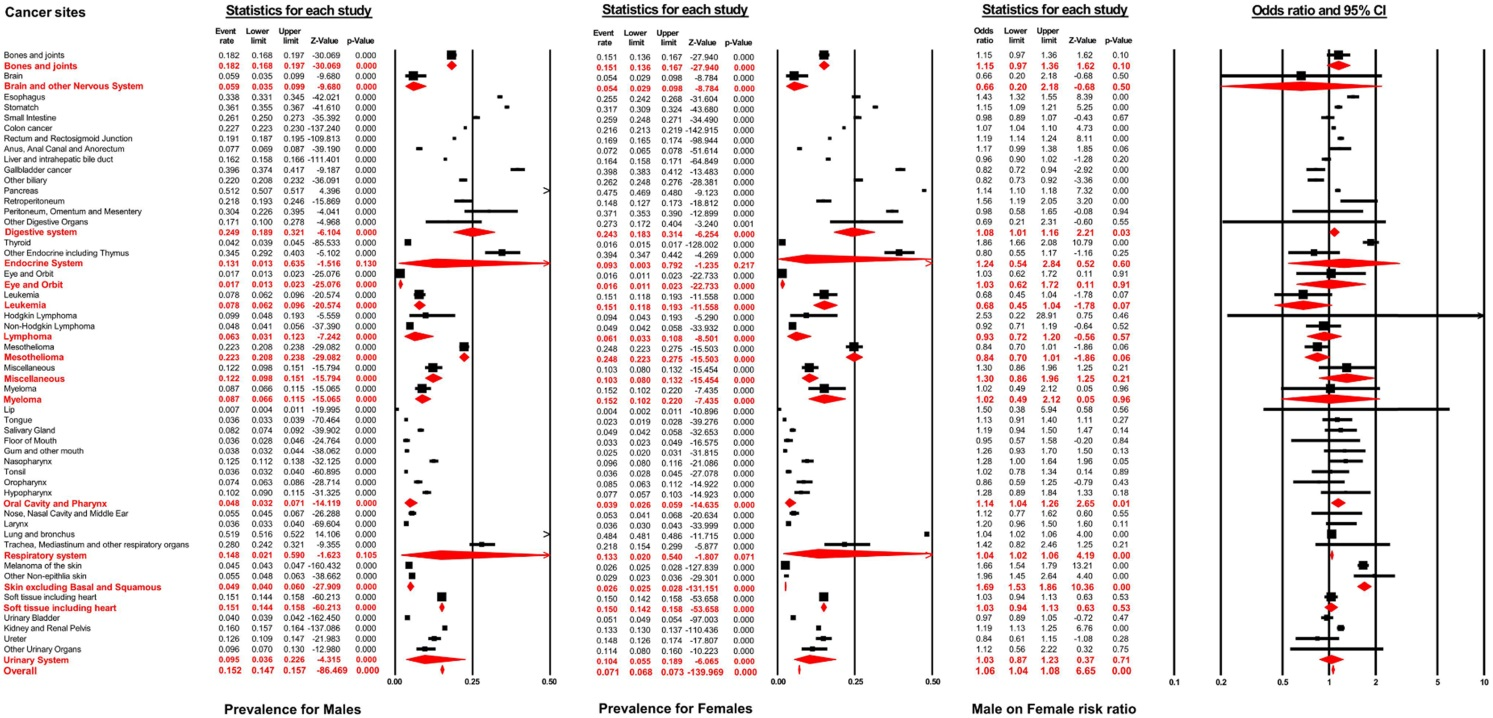
Forest plot for the pooled prevalence of metastasis by sex and the effect of male sex on the development of metastasis for different non-sex-specific cancer systems.

As displayed in **Figure 2**, the distant metastasis prevalence across all non-sex-specific cancers for males ranged from 0.7% (95% CI: 0.4-1.1%, lip) to 51.9% (95% CI: 51.6-52.2%, lung and bronchus). Similarly, the pooled prevalence varied between 0.4% (95% CI: 0.2-1.1%, lip) to 48.4% (95% CI: 48.1-48.6%, lung and bronchus) for female patients. In general, the distant metastasis pooled prevalence for male and female patients was 15.2% (95% CI: 14.7-15.7%) and 7.1% (95% CI: 6.8-7.3%), respectively, across all non-sex-specific cancers. Interestingly, the inconsistent pooled prevalence was observed for different cancer systems, when analyzed according to sex type. Specifically, the digestive system showed the highest pooled prevalence of distant metastasis in males (24.9%, 95% CI: 18.9-32.1%), while mesothelioma exhibited the highest prevalence (24.8%, 95% CI: 22.3-27.5%) in female patients. Conversely, the eye and orbit displayed the lowest prevalence amongst both male and female patients (1.7%, 95% CI: 1.3-2.3% for male and 1.6%, 95% CI: 1.1-2.3% for female).

As suggested in **Figure 4**, the prevalence of distant metastasis displayed an age-dependent characteristic. Specifically, when patients' age rose from 0 to 30 years, the pooled prevalence of distant metastasis for both males and females decreased significantly to the lowest. Subsequently, the prevalence of metastasis for males and females showed an increasing trend with the rise in patients' age, reaching a peak at the age between 71 and 80 years. Following this period, amongst both sexes, the prevalence rate dropped with the increase in age. The distant metastasis prevalence was higher in males than in females for patients at the age before 61 to 70 years, while the prevalence of distant metastasis was lower in males than in female patients aged over 70 years. The male-to-female prevalence ratio increased remarkably with age, reaching its peak from 0 to 30 years. Within the 31 to 70 year age-period, the aging process appeared to affect the male-to-female prevalence ratio, which decreased markedly to approximately 1.00. Subsequently, the male-to-female prevalence ratio plateaued at around 0.90 from age over 70 years.

According to multivariable logistic regression, the male sex effect on the development of distant metastasis varied according to cancer sites. Male sex represented an independent metastatic risk factor in other non-epithelia skin cancer (OR=1.96, 95% CI: 1.45-2.64; *P<*0.01), in thyroid cancer (OR=1.86, 95% CI: 1.66-2.08; *P<*0.01) and in melanoma (OR=1.66, 95% CI: 1.54-1.79; *P<*0.01). In contrast, male sex showed a protective effect in gallbladder cancer (OR=0.82, 95% CI: 0.72-0.94; *P<*0.01) and other biliary cancer (OR=0.82, 95% CI: 0.73-0.94; *P<*0.01). Nevertheless, the pooled meta-analysis results demonstrated that male sex still represented an independent risk factor for developing distant metastasis in digestive systems (pooled OR=1.08, 95% CI: 1.01-1.16; *P<*0.01), the oral cavity and pharynx (pooled OR=1.14, 95% CI: 1.04-1.26; *P<*0.01), the respiratory system (pooled OR=1.04, 95% CI: 1.02-1.06; *P<*0.01), and in skin, excluding basal and squamous cell, (pooled OR=1.69, 95% CI: 1.53-1.86; *P<*0.01). After combining all non-sex-specific cancers, male patients appeared to have a significantly higher occurrence of distant metastasis than females (pooled OR=1.06, 95% CI: 1.04-1.08; *P<*0.01) (see **Figure 2**).

Furthermore, as showed in Table S1, when stratified by age groups (age <65 years and age ≥65 years), males were still more likely to develop distant metastasis (pooled OR=1.23, 95% CI: 1.27-1.29; *P<*0.01 for age <65 years; pooled OR=1.04, 95% CI: 1.02-1.06; *P<*0.01 for age ≥65 years). The sex differences among patients aged less than 65 years were more significant than patients aged 65 years or older (*P<*0.01).

Table S2 shows the analysis performed according to different ethnic groups. Male gender was significantly associated with the risk for distant metastasis among all race groups (pooled OR=1.07, 95% CI: 1.05-1.09; *P<*0.01 for white; pooled OR=1.08, 95% CI: 1.04-1.12;* P<*0.01 for black; pooled OR=1.16, 95% CI: 1.00-1.33;* P=*0.045 for American Indian or Alaska native; pooled OR=1.13, 95% CI: 1.04-1.23;* P<*0.01 for Asian or Pacific Islander) (see Table S2). There was no statistically significant difference amongst all four ethnic groups (*P*=0.43).

### Sex disparities in the prognosis of metastatic cancer

**Figure 3:**
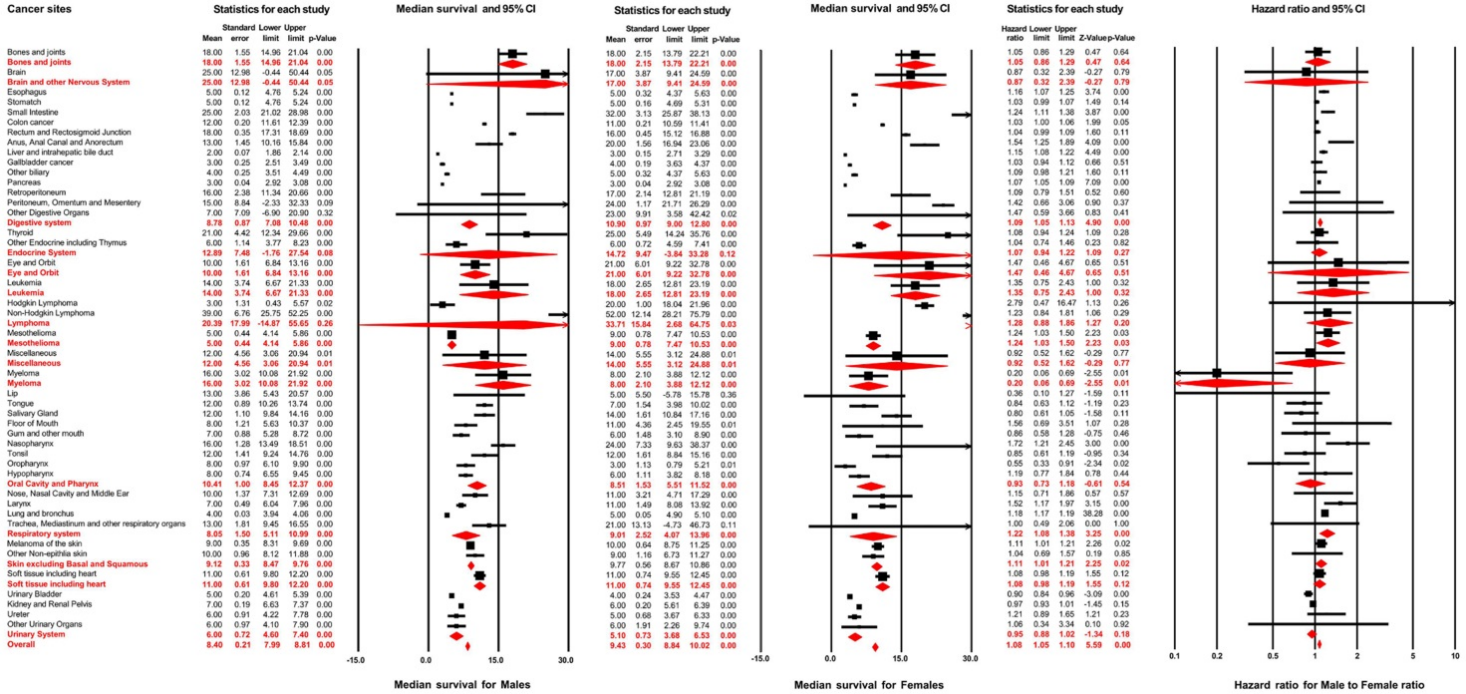
Forest plot for the prognosis of metastasis and the effect of male sex on it across different non-sex-specific cancer types.

**Figure 4:**
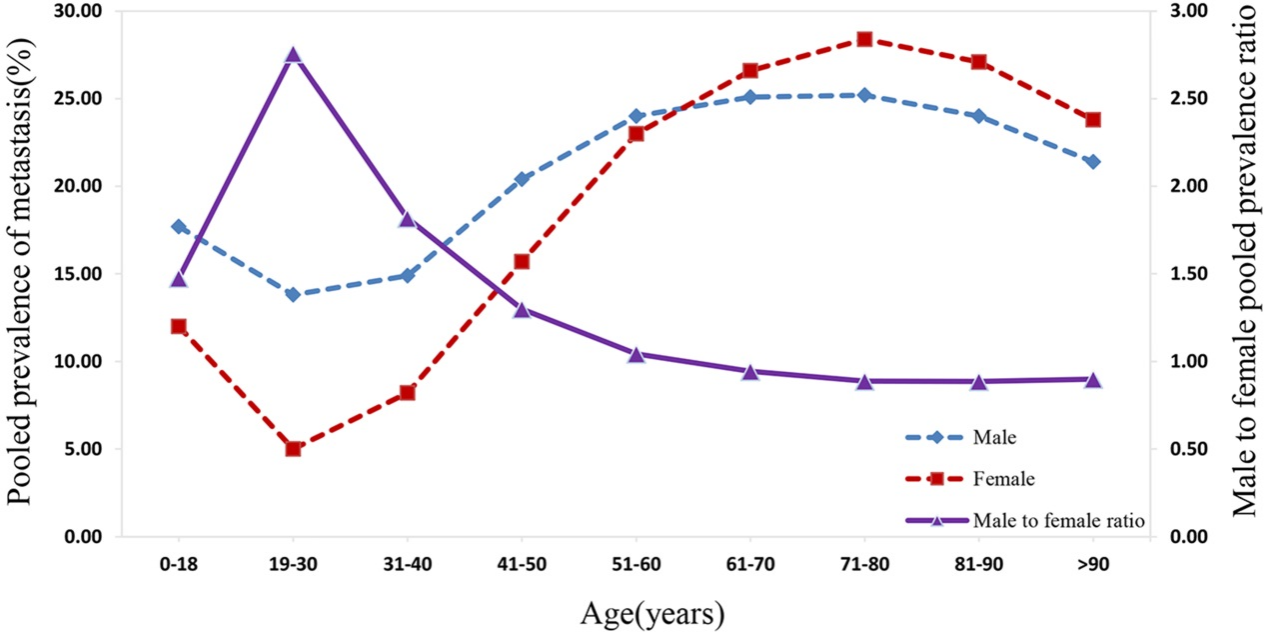
The pooled male and female prevalence of metastasis and the male-to-female prevalence ratio across different age groups.

A total of 279,573 patients developed distant metastasis. Amongst these, male and female patients accounted for 56.3% and 43.7%, respectively. **Figure 3** illustrates the median survival time of male patients: this outcome was 8.40 months (95% CI: 7.99-8.81) across all non-sex-specific cancers, ranging from 2.00 months (95% CI: 1.86-2.14 for liver and intrahepatic bile duct) to 39.00 months (95% CI: 25.75-52.25 for Non-Hodgkin lymphoma) for each cancer site. The female patients' analysis demonstrated that the pooled median survival time was 9.40 months (95% CI: 8.84-10.02). Furthermore, for each cancer site in female patients, the median survival time varied from 3.00 months (95% CI: 0.79-5.21, for oropharynx) to 52.00 months (95% CI: 28.21-75.79, for Non-Hodgkin lymphoma).

Results of multivariable Cox regression are displayed in **Figure 3.** The hazard ratio of male sex varied from 0.55 (95% CI: 0.33-0.90; *P*=0.02, for Oropharynx) to 1.72 (95% CI: 1.21-2.46; *P*<0.01, for Nasopharynx) for the overall survival of distant metastasis in different cancer sites. The pooled results, from meta-analysis for different cancer systems, suggested that male sex was negatively associated with the overall survival after distant metastasis in digestive systems (pooled HR=1.09, 95% CI: 1.05-1.13; *P*<0.01), mesothelioma (pooled HR=1.24, 95% CI: 1.03-1.50; *P*=0.03), respiratory system (pooled HR=1.22, 95% CI: 1.08-1.38; *P*<0.01) and skin, excluding basal and squamous cell (pooled HR=1.11, 95% CI: 1.01-1.21; *P*=0.02). After combining all non-sex-specific cancers, male patients displayed a significantly reduced overall survival outcome after distant metastasis compared with females (pooled HR=1.08, 95% CI: 1.05-1.10; *P*<0.01).

As showed in Table S3, when stratified by age groups (age <65 years and age ≥65 years), male sex was significantly associated with worse overall survival after distant metastasis (pooled HR=1.18, 95% CI: 1.16-1.20; *P<*0.01 for age <65 years; pooled HR=1.06, 95% CI: 1.02-1.10; *P<*0.01 for age ≥65 years). The effect of male sex on patients' aged younger than 65 years was higher than the patients aged 65 years or older (*P<*0.01).

Comparison amongst different ethnic groups supported a similar conclusion, as demonstrated in Table S4: male gender was associated with an adverse prognosis after distant metastasis among all race groups (pooled HR=1.06, 95% CI: 1.03-1.09; *P<*0.01 for white; pooled HR =1.07, 95% CI: 1.03-1.11;* P<*0.01 for black; pooled HR =1.21, 95% CI: 1.16-1.23;* P<*0.01 for Asian or Pacific Islander). Patients of American Indian or Alaska native race were excluded from this analysis due to the limited sample size. The sex differences were higher amongst Asian or Pacific Islander patients than in patients from other ethnic groups (*P*<0.01).

## Discussion

Based on data representing around 34.6% of the U.S. population, the gender discrepancies in distant metastasis occurrence and overall survival after metastasis were evaluated in this study. The main finding was that male patients showed higher risks for distant metastasis occurrence and worse overall survival than females for the majority of cancer types. Consistent pooled results were observed across all non-sex-specific cancers. The effects of male sex on both the occurrence and prognosis of distant metastasis were greater in patients younger than 65 years. Moreover, the male-to-female hazard ratio for overall survival after metastasis was significantly higher in Asian or Pacific Islander populations than other ethnic groups. Additionally, the pooled prevalence of distant metastasis increased considerably after the age of 30 years and dropped slightly after the age of 70 years.

The effects of male sex varied for different cancer types. Consistent with previous studies, females with esophageal cancer or pancreatic cancer were protected against metastasis and had a more favorable subsequent survival 15,16,27-29. But inconsistent patterns were observed for gastric cancer. Data from Swedish cancer registers suggested no gender impact on either distant metastasis occurrence or prognosis, while in our study, a positive relationship between male sex and the metastatic risk was reported 30. Prior studies suggested that females were less likely to develop distant metastasis in either colon or rectal cancer, and female patients younger than 45 years had a survival advantage for metastatic colorectal cancer 12,31,32. However, as the age of female patients increased, these differences diminished until they became non-significant 12. Similar risk and survival trends were observed for males and females in our analyses of the colon and rectal cancer, as the sex discrepancies presented when patients were younger than 65 years.

In this study, female patients had a decreased risk of developing distant metastasis in melanoma, consistent with a previous report 14. Concerning prognosis, a sex effect on death due to melanoma was only observed in localized or regional disease (data from the SEER database 1992-2011) 33. In contrast, this study (data from the SEER database 2010-2016) found a slight difference in overall survival between male and female patients with metastatic melanoma. Based on our analysis, carcinoma of the lung and bronchus was associated with lower risks of distant metastasis occurrence and worse prognosis in males compared with females. Consistent trends were found in other studies, and notably, EGFR inhibitors exhibited better performance in treating female patients with lung cancer than male patients 34. A series of studies assessed the effect of gender on the risk of distant metastasis in thyroid carcinoma 17,35,36. Female sex was found to be a protective factor for distant metastasis in our study, consistent with an earlier meta-analysis of 29 studies 17. As previously reported, male patients were more likely to have metastatic spread in laryngeal cancer and renal cancer, while a survival advantage in males was found in metastatic bladder cancer in this study 37-39.

A higher risk for distant metastasis occurrence was also observed in male patients with other non-epithelial skin cancers and retroperitoneal cancer, while male sex had a protective effect on the risk for metastatic gallbladder cancer and other biliary cancers. In addition, a female survival advantage was observed in nasopharyngeal carcinoma, mesothelioma, liver cancer, and carcinoma of the anus, anal canal, and anorectum after distant metastasis, whereas male patients had a more favorable prognosis in oropharyngeal cancer and myeloma. These gender discrepancies were first identified in our study.

Interpreting the sex disparities in distant metastasis occurrence and prognosis is more challenging because of the necessity to consider multiple potential factors and their interactions. Lower health awareness, less health care utilization, and fewer preventive health behaviors in males might lead to diagnostic delays 4,40,41. These differences might explain why male patients had worse clinical characteristics than females at diagnosis, including higher tumor differentiated grade, advanced tumor stage, and nodal stage, as reported in this study (**Table 1**) and consistently found throughout previous publications 33,42. More comorbid conditions at diagnosis, greater use of tobacco, and higher alcohol consumption in males might also contribute to a higher risk for distant metastasis occurrence and worse subsequent survival 4,43-45.

There is also growing evidence that the hormone axis that distinguishes males from females might influence the progression of non-sex-specific cancers, as measured by tumor grade, lymphatic vessel invasion, proliferation index, and mutation status 2,4. Previous publications have suggested that the expression of estrogen receptors is related to the clinical features of gastric cancer by regulating the growth and proliferation of gastric cancer cells 46-48. Meanwhile, positive expression of estrogen and progesterone receptors might contribute to an earlier tumor stage, higher histologic differentiation, and a more favorable prognosis in lung cancer patients 2,49.

Different molecular characterizations between male and female patients are receiving increased global attention. Biallelic expression of “escape from X-inactivation tumor-suppressor” (EXITS) genes in females might reduce the risk of complete functional loss of X chromosomes caused by a single mutation 50,51. Therefore, this discrepancy could partially explain the lower risk of metastasis in females across many cancers. Additionally, the interactions between sex chromosomes and the level of sex hormones might also have an impact on cell metabolism and the immune response of patients 52. Comparing to males, females generally display greater innate and adaptive immune responses, leading to more rapid elimination of pathogens and lower tumor susceptibility 53. Gender-related signatures were exhibited in up to 53% of clinically actionable genes 1. These sex discrepancies in molecular patterns might influence the efficacy and toxicity of chemotherapy, as well as the clinical outcomes of patients 2,26. The rates of EGFR-sensitizing mutations, which were found to be higher in females than males, were associated with improved survival in metastatic non-small-cell lung cancer (NSCLC) patients 34,54. This reflected the sex difference in therapeutic response to EGFR inhibitors. Among patients with colon cancer, gender played a pivotal role in genetic polymorphisms in drug-associated clinically actionable genes (XPD, MTHFR, and ECCR1 genes), leading to different toxic responses to chemotherapies 55.

To our knowledge, this is the first study to systematically investigate sex discrepancies in metastatic risk and subsequent prognosis across non-sex-specific cancers. Our results confirm the hypothesis that female patients are less likely to establish metastases at distant sites, such that females present a better subsequent prognosis for the vast majority of cancer types. Consistent trends were observed in age- and race-stratified analyses. These findings highlight the importance of conducting sex-specific distant metastasis screening, prevention, and treatment. Therefore, male patients should be provided with more health education and services, and they could also be selected as potential candidates for more frequent screening for distant metastasis. Meanwhile, as the important roles of sex hormones in the development and treatment of cancers have been reported in previous studies 56-61, it is a promising way to treat males and females as biologically different groups for the prevention and treatment of cancer development and progression 52. Moreover, sex disparities may also exist in the outcomes of surgical treatment, radiotherapy, and modality treatments, and further investigation is required to explore the most effective treatment strategy for each sex 52.

There are several limitations in this study. A few factors that have been recognized as independently associated with cancer progression were not included, such as tobacco and alcohol consumption and the comorbidity burden. The outcome reported in our study is the overall survival after distant metastasis, although eligible patients may have died from comorbidities other than the target cancer. Further research should evaluate the gender effects on cancer-specific death. Significant heterogeneities were found in the meta-analysis, and thus interpretations of the combined results should be made with caution. The present study was performed based on the patients' characteristics at admission, and all the metastatic patients included in this study were synchronous metastasis. However, the record of the occurrence of metachronous metastasis during follow-up was not available in the database, and thus the results might be partially affected. Further comparison of the synchronous and metachronous metastasis should be valuable and be conducted in the future with available data. Additionally, this study was conducted using a single database with a large sample size, external validation with another national database or external cohort, and comprehensive meta-analysis could be applied to improve credibility.

## Conclusion

Male cancer patients present worse clinical characteristics than female patients at diagnosis. Moreover, the male gender is an independent risk factor associated with the occurrence and prognosis of synchronous distant metastasis. Thus, more cancer screening opportunities and health care services should be provided to male subjects. Furthermore, the results of the present study imply that individualized treatments should take sex differences into account in the future.

## Supplementary Material

Supplementary tables.

## Figures and Tables

**Table 1:** Demographic and clinical characteristics distribution of the included patients

Factors	Male		Female		χ^2^/Z	*P*
	N	%	N	%		
All patients	660473	100.0	519895	100.0		
**Age (years)**					2.16	0.14
<65	317775	48.1	249430	48.0		
≥65	342698	51.9	270465	52.0		
**Race**					419.41	<0.01
White	536696	81.2	414932	79.8		
Black	62885	9.5	54330	10.5		
Asian or Pacific Islander	46637	7.1	39126	7.5		
American Indian/Alaska Native	4383	0.7	3581	0.7		
Unknown	9872	1.5	7926	1.5		
**Marital status**					28228.13	<0.01
Married	381959	57.8	223701	43.0		
Unmarried	221425	33.5	250953	48.3		
Unknown	57089	8.7	45241	8.7		
**Insurance status**					3.29*	<0.01
Uninsured	20634	3.1	13388	2.6		
Any Medicaid	82670	12.5	69923	13.4		
Insured	517499	78.4	405304	78.0		
Unknown	39670	6.0	31280	6.0		
**Income**					17.98*	<0.01
<6000	153160	23.2	112545	21.7		
6000-7000	193291	29.3	153399	29.5		
7000-8000	98248	14.9	79012	15.2		
>8000	215716	32.6	174894	33.6		
Unknown	58	0.0	45	0.0		
**Differentiated Grade**					22.25*	<0.01
Grade I	48898	7.4	49801	9.6		
Grade II	164702	24.9	120799	23.2		
Grade III	116273	17.6	74174	14.3		
Grade IV	41031	6.2	21618	4.1		
Unknown	289569	43.9	253503	48.8		
**T Stage**					4.48*	<0.01
T1	232321	35.2	184676	35.5		
T2	123220	18.7	97284	18.7		
T3	142025	21.5	110838	21.3		
T4	82623	12.5	64773	12.5		
Unknown	80284	12.1	62324	12.0		
**N stage**					11.55*	<0.01
N0	400769	60.7	320435	61.6		
N1	92978	14.1	75655	14.6		
N2	90113	13.6	63639	12.3		
N3	25421	3.8	18390	3.5		
Unknown	51192	7.8	41776	8.0		
**M stage**					19.61	<0.01
M0	503023	76.2	397772	76.5		
M1	157450	23.8	122123	23.5		
**Liver metastasis**					27.91	<0.01
Yes	62216	9.4	47692	9.2		
No	569983	86.3	449369	86.4		
Unknown	28274	4.3	22834	4.4		
**Lung metastasis**					49.19	<0.01
Yes	45452	6.9	34175	6.6		
No	584566	88.5	461203	88.7		
Unknown	30455	4.6	24517	4.7		
**Bone metastasis**					567.59	<0.01
Yes	42166	6.4	27802	5.4		
No	589286	89.2	468467	90.1		
Unknown	29021	4.4	23626	4.5		
**Brain metastasis**					58.51	<0.01
Yes	20781	3.1	17539	3.4		
No	610092	92.4	478456	92.0		
Unknown	29600	4.5	23900	4.6		
**Surgery**					2120.31	<0.01
No	282387	42.7	200536	38.6		
Yes	373636	56.6	315912	60.8		
Unknown	4450	0.7	3447	0.6		
**Radiation therapy**					306.96	<0.01
No	491905	74.5	394369	75.9		
Yes	164012	24.8	121868	23.4		
Unknown	4556	0.7	3658	0.7		
**Chemotherapy**					3437.37	<0.01
No	435352	65.9	369020	71.0		
Yes	225121	34.1	150875	29.0		
**Malignancy system**					NA	NA
Oral Cavity and Pharynx	41250	6.3	16256	3.1		
Digestive System	234234	35.5	187247	36.0		
Respiratory System	154553	23.4	132710	25.5		
Bones and joints	2693	0.4	2039	0.4		
Soft tissue including Heart	9475	1.4	7501	1.4		
Skin excluding Basal and Squamous Cell	67713	10.3	51516	9.9		
Urinary System	120453	18.2	53894	10.4		
Eye and Orbit	2278	0.3	1932	0.4		
Brain and other Nervous System	220	0.0	184	0.0		
Endocrine System	18902	2.9	61650	11.9		
Lymphoma	3496	0.5	2871	0.6		
Myeloma	517	0.1	145	0.0		
Leukemia	965	0.1	350	0.1		
Mesothelioma	3126	0.5	1048	0.2		
Miscellaneous	598	0.1	552	0.1		

*Ordinal categorical variables were compared using the rank-sum test.

## Abbreviations

SEER: Surveillance, Epidemiology, and End Results
OR: Odds ratio
HR: Hazard ratio
CI: Confidence interval
EGFR: Epidermal growth factor receptor
SE: Standard error
