# Supplementary material for: Sex Disparities in the Clinical Characteristics, Synchronous Distant Metastasis Occurrence and Prognosis: A Pan-cancer Analysis
Source: J Cancer. 2021 Jan 1;12(2):498–507. doi: 10.7150/jca.50536 (PMC7739003; doi:10.7150/jca.50536)
Supplement: Supplementary file 1 — Supplementary tables. [file jcav12p0498s1.pdf]

Table S1. The pooled prevalence of metastasis by sex and the effect of male sex on the development of metastasis for different non-sex-specific cancers among different age groups.

Cancer Site	Age<65						Age≥65					
	Risk of M-Met						Risk of M-Met					
	Male		Female		OR (95%CI) [a]	*P*-value	Male		Female		OR (95%CI) [a]	*P*-value
	case (N)	Event rate	case (N)	Event rate			case (N)	Event rate	case (N)	Event rate		
**Oral Cavity and Pharynx**	1202	0.046	312	0.035	1.11(0.94-1.30)	0.23	826	0.055	285	0.038	1.24(1.07-1.44)	0.01
Lip	8	0.007	1	0.003	2.41(0.11-53.38)	0.58	8	0.006	3	0.005	1.41(0.29-6.94)	0.67
Tongue	279	0.033	53	0.019	1.21(0.89-1.65)	0.23	198	0.041	60	0.028	1.15(0.84-1.57)	0.38
Salivary Gland	131	0.075	61	0.039	1.15(0.81-1.62)	0.43	167	0.089	67	0.064	1.32(0.96-1.81)	0.09
Floor of Mouth	39	0.036	14	0.036	0.89(0.46-1.72)	0.73	20	0.036	11	0.031	1.04(0.45-2.40)	0.93
Gum and other mouth	80	0.039	29	0.024	1.09(0.69-1.74)	0.72	64	0.036	48	0.025	1.55(1.03-2.33)	0.04
Nasopharynx	219	0.117	78	0.104	1.11(0.83-1.47)	0.47	91	0.149	20	0.075	1.96(1.14-3.36)	0.02
Tonsil	225	0.030	37	0.029	0.92(0.64-1.32)	0.65	130	0.054	33	0.047	1.11(0.74-1.65)	0.61
Oropharynx	82	0.069	26	0.087	0.77(0.48-1.26)	0.29	57	0.083	17	0.082	1.07(0.59-1.95)	0.82
Hypopharynx	139	0.109	13	0.048	2.33(1.28-4.23)	0.01	91	0.092	26	0.110	0.87(0.53-1.43)	0.58
**Digestive system**	33534	0.282	21116	0.268	1.07(0.98-1.17)	0.11	30697	0.267	28235	0.260	1.07(0.98-1.17)	0.14
Esophagus	3123	0.385	520	0.316	1.28(1.12-1.45)	<0.01	2798	0.298	644	0.220	1.61(1.44-1.79)	<0.01
Stomach	4363	0.417	2351	0.374	1.12(1.04-1.20)	<0.01	3940	0.314	2378	0.275	1.21(1.13-1.29)	<0.01
Small Intestine	869	0.269	716	0.260	1.01(0.89-1.11)	0.88	700	0.252	741	0.258	0.94(0.83-1.08)	0.32
Colon cancer	8419	0.249	7198	0.250	0.95(0.91-0.99)	0.02	7724	0.206	8442	0.193	1.17(1.12-1.22)	<0.01
Rectum and Rectosigmoid Junction	4605	0.200	2617	0.165	1.24(1.17-1.32)	<0.01	2571	0.178	2041	0.175	1.11(1.04-1.20)	<0.01
Anus, Anal Canal and Anorectum	184	0.077	254	0.071	1.18(0.95-1.46)	0.13	87	0.077	183	0.073	1.15(0.87-1.53)	0.33
Liver and intrahepatic bile duct	3394	0.163	860	0.169	0.93(0.85-1.01)	0.10	2100	0.161	1041	0.161	0.96(0.88-1.04)	0.34

Gallbladder cancer	301	0.444	652	0.424	0.94(0.76-1.16)	0.57	475	0.370	1094	0.383	0.76(0.64-0.89)	<0.01
Other biliary	459	0.253	351	0.287	0.78(0.65-1.94)	0.01	579	0.199	663	0.250	0.79(0.69-0.91)	<0.01
Pancreas	7650	0.540	5165	0.490	1.15(1.09-1.22)	<0.01	9646	0.492	10333	0.467	1.12(1.07-1.17)	<0.01
Retroperitoneum	138	0.246	81	0.147	1.69(1.19-2.39)	<0.01	60	0.173	55	0.151	1.25(0.80-1.96)	0.33
Peritoneum, Omentum and Mesentery	23	0.359	344	0.352	1.13(0.56-2.27)	0.73	11	0.229	612	0.383	0.90(0.39-2.06)	0.80
Other Digestive Organs	6	0.171	7	0.280	0.53(0.15-1.84)	0.32	6	0.171	8	0.267	0.95(0.11-7.81)	0.96
Respiratory system	28731	0.497	21998	0.497	1.18(0.98-1.43)	0.09	44379	0.459	40336	0.456	1.04(1.02-1.06)	<0.01
Nose, Nasal Cavity and Middle Ear	50	0.056	24	0.051	1.10(0.65-1.86)	0.72	40	0.054	30	0.055	1.21(0.71-2.08)	0.49
Larynx	248	0.037	50	0.030	1.39(1.00-1.91)	0.046	221	0.035	61	0.044	0.99(0.73-1.34)	0.95
Lung and bronchus	28305	0.568	21901	0.521	1.06(1.03-1.09)	<0.01	44110	0.492	40241	0.465	1.04(1.02-1.06)	<0.01
Trachea, Mediastinum and other respiratory organs	128	0.296	23	0.209	1.59(0.96-2.64)	0.07	8	0.148	4	0.286	0.15(0.01-3.49)	0.17
Bones and joints	412	0.182	258	0.154	1.11(0.92-1.33)	0.27	79	0.183	49	0.133	1.34(0.86-2.09)	0.20
Soft tissue including heart	988	0.159	764	0.153	1.04(0.93-1.17)	0.50	440	0.136	360	0.143	0.94(0.80-1.11)	0.46
Skin excluding Basal and Squamous Cell	1565	0.044	682	0.021	1.93(1.74-2.13)	<0.01	1520	0.047	672	0.036	1.63(1.10-2.42)	0.01
Melanoma of the skin	1515	0.044	663	0.021	1.93(1.74-2.13)	<0.01	1374	0.046	620	0.036	1.38(1.23-1.54)	<0.01
Other Non-epithelia skin	50	0.045	19	0.026	1.79(0.98-3.25)	0.06	146	0.060	52	0.030	2.07(1.47-2.94)	<0.01
Urinary System	5383	0.101	2051	0.086	1.15(0.89-1.49)	0.27	5736	0.083	3307	0.106	0.99(0.88-1.12)	0.89
Urinary Bladder	936	0.043	348	0.053	0.96(0.83-1.11)	0.58	1794	0.039	820	0.051	0.95(0.86-1.05)	0.31
Kidney and Renal Pelvis	4362	0.148	1646	0.105	1.30(1.20-1.40)	<0.01	3812	0.178	2371	0.164	1.08(1.01-1.15)	0.02
Ureter	42	0.149	24	0.157	1.32(0.59-2.94)	0.50	106	0.119	98	0.146	0.81(0.58-1.15)	0.23
Other Urinary Organs	13	0.084	10	0.092	1.60(0.38-6.78)	0.52	24	0.103	18	0.131	0.78(0.31-1.93)	0.59
Eye and Orbit	25	0.017	19	0.016	1.07(0.55-2.06)	0.84	14	0.017	12	0.017	0.97(0.42-2.25)	0.94
Brain and other Nervous System	5	0.057	4	0.047	1.07(0.11-10.12)	0.95	8	0.061	6	0.061	1.63(0.28-9.55)	0.59
Brain	5	0.057	4	0.047	1.07(0.11-10.12)	0.95	8	0.061	6	0.061	1.63(0.28-9.55)	0.59
Endocrine System	497	0.034	554	0.011	1.52(0.64-3.63)	0.34	378	0.089	590	0.061	1.42(1.20-1.68)	0.85

**Thyroid**	**425**	**0.029**	**441**	**0.009**	**2.31(1.99-2.68)**	**<0.01**	**353**	**0.085**	**542**	**0.057**	**1.42(1.20-1.67)**	**<0.01**
**Other Endocrine including Thymus**	**72**	**0.375**	**113**	**0.401**	**0.95(0.61-1.50)**	**0.82**	**25**	**0.281**	**48**	**0.378**	**0.51(0.25-1.07)**	**0.51**
**Lymphoma**	**68**	**0.038**	**53**	**0.036**	**1.00(0.67-1.49)**	**0.98**	**104**	**0.062**	**91**	**0.065**	**0.88(0.64-1.21)**	**0.45**
**Hodgkin Lymphoma**	**3**	**0.081**	**4**	**0.129**	**0.60(0.12-2.89)**	**0.53**	**4**	**0.118**	**2**	**0.061**	**2.07(0.35-12.13)**	**0.42**
**Non-Hodgkin Lymphoma**	**65**	**0.037**	**49**	**0.034**	**1.03(0.68-1.56)**	**0.89**	**100**	**0.060**	**89**	**0.065**	**0.86(0.62-1.18)**	**0.36**
**Myeloma**	**15**	**0.098**	**7**	**0.179**	**0.93(0.14-6.17)**	**0.94**	**30**	**0.082**	**15**	**0.142**	**1.07(0.44-2.60)**	**0.88**
**Leukemia**	**24**	**0.085**	**23**	**0.193**	**0.73(0.34-1.57)**	**0.42**	**51**	**0.075**	**30**	**0.130**	**0.62(0.35-1.07)**	**0.09**
**Mesothelioma**	**196**	**0.277**	**100**	**0.270**	**0.86(0.63-1.18)**	**0.35**	**500**	**0.207**	**160**	**0.236**	**0.84(0.68-1.05)**	**0.12**
**Miscellaneous**	**32**	**0.145**	**28**	**0.127**	**1.20(0.65-2.23)**	**0.56**	**41**	**0.109**	**29**	**0.088**	**1.31(0.73-2.34)**	**0.36**
**Total**	**72677**	**0.228**	**47969**	**0.191**	**1.23(1.17-1.29)**	**<0.01**	**84803**	**0.247**	**74177**	**0.274**	**1.04(1.02-1.06)**	**<0.01**

[a] Adjusted for all confounders: race, marital status, income, insurance, differentiated grade, T stage, and N stage.

Table S2. The pooled prevalence of metastasis by sex and the effect of male sex on the development of metastasis for different non-sex-specific cancers among different race groups.

Cancer Site	White						Black						American Indian/Alaska						Asian					
	Risk of M-Met						Risk of M-Met						Risk of M-Met						Risk of M-Met					
	Male		Female		OR (95%CI) [a]	*P*-value	Male		Female		OR (95%CI) [a]	*P*-value	Male		Female		OR (95%CI) [a]	*P*-value	Male		Female		OR (95%CI) [a]	*P*-value
	case (N)	Event rate	case (N)	Event rate			case (N)	Event rate	case (N)	Event rate			case (N)	Event rate	case (N)	Event rate			case (N)	Event rate	case (N)	Event rate		
**Oral Cavity and Pharynx**	1510	0.044	413	0.032	1.16(1.03-1.30)	0.02	286	0.083	98	0.064	0.98(0.75-1.27)	0.88	17	0.060	9	0.075	0.61(0.21-1.74)	0.36	206	0.073	64	0.042	1.37(0.98-1.92)	0.06
Lip	14	0.006	4	0.005	1.29(0.32-5.14)	0.72	0	0.000	0	0.000	-	-	0	0.000	0	0.000	-	-	2	0.077	0	0.000	-	-
Tongue	406	0.035	91	0.023	1.11(0.87-1.41)	0.42	51	0.060	2	0.006	1.32(0.67-2.61)	0.42	2	0.028	1	0.036	0.78(0.07-8.99)	0.84	16	0.027	9	0.021	1.08(0.41-2.83)	0.88
Salivary Gland	253	0.085	100	0.051	1.21(0.94-1.57)	0.32	25	0.092	19	0.064	1.16(0.53-2.51)	0.71	2	0.125	2	0.125	1.00(0.12-8.13)	1.00	17	0.059	7	0.027	2.03(0.68-6.08)	0.21
Floor of Mouth	42	0.032	19	0.030	0.85(0.48-1.53)	0.60	14	0.064	5	0.063	0.94(0.27-3.27)	0.92	0	0.000	1	0.111	-	-	3	0.059	0	0.000	-	-
Gum and other mouth	108	0.037	56	0.023	1.30(0.91-1.84)	0.15	26	0.068	12	0.038	1.48(0.69-3.20)	0.32	3	0.107	0	0.000	-	-	6	0.016	8	0.033	0.72(0.15-3.36)	0.67
Nasopharynx	134	0.117	33	0.073	1.54(1.02-2.32)	0.04	38	0.134	19	0.151	0.85(0.45-1.60)	0.62	5	0.147	5	0.278	0.45(0.11-1.82)	0.27	130	0.131	39	0.094	1.39(0.94-2.06)	0.10
Tonsil	293	0.034	54	0.033	1.07(0.79-1.44)	0.68	45	0.057	16	0.082	0.64(0.34-1.21)	0.17	2	0.029	0	0.000	-	-	13	0.044	0	0.000	-	-
Oropharynx	97	0.063	28	0.070	0.86(0.55-1.36)	0.52	35	0.137	14	0.167	0.73(0.34-1.53)	0.40	0	0.000	0	0.000	-	-	7	0.137	1	0.063	2.39(0.27-21.02)	0.43
Hypopharynx	163	0.095	28	0.074	1.25(0.81-1.91)	0.32	52	0.138	11	0.117	1.20(0.55-2.60)	0.64	3	0.115	0	0.000	-	-	12	0.081	0	0.000	-	-
**Digestive system**	49452	0.276	37080	0.263	1.07(1.00-1.16)	0.054	8440	0.304	7203	0.288	1.18(1.08-1.28)	<0.01	597	0.292	394	0.251	1.35(1.11-1.63)	<0.01	5542	0.243	4522	0.253	1.05(0.93-1.20)	0.44
Esophagus	5084	0.340	918	0.257	1.44(1.31-1.58)	<0.01	512	0.339	178	0.261	1.38(1.11-1.72)	<0.01	49	0.389	12	0.333	1.39(0.45-4.29)	0.57	260	0.309	53	0.212	1.50(1.03-2.19)	0.03
Stomach	6100	0.368	3185	0.322	1.17(1.10-1.24)	<0.01	1111	0.382	673	0.297	1.33(1.16-1.51)	<0.01	98	0.439	53	0.351	1.30(0.78-1.26)	0.30	955	0.304	787	0.324	0.94(0.82-1.07)	0.32
Small Intestine	1255	0.269	1104	0.263	1.01(0.91-1.11)	0.92	231	0.258	268	0.251	1.03(0.82-1.29)	0.80	9	0.257	4	0.200	0.40(0.06-2.94)	0.36	70	0.212	78	0.293	0.49(0.32-0.76)	<0.01
Colon cancer	12176	0.221	11596	0.210	1.07(1.03-1.11)	<0.01	2519	0.282	2624	0.263	1.08(1.00-1.17)	0.04	126	0.231	124	0.213	1.13(0.81-1.58)	0.47	1282	0.214	1250	0.209	1.09(0.98-1.20)	0.11
Rectum and Rectosigmoid Junction	5558	0.193	3633	0.176	1.15(1.10-1.21)	<0.01	849	0.220	537	0.165	1.39(1.22-1.58)	<0.01	83	0.238	51	0.195	1.51(0.98-2.34)	0.06	665	0.168	424	0.150	1.22(1.06-1.42)	0.01
Anus, Anal Canal and Anorectum	212	0.076	363	0.068	1.21(1.00-1.46)	0.048	39	0.072	47	0.086	0.89(0.54-1.46)	0.64	4	0.143	3	0.094	1.61(0.33-7.91)	0.56	15	0.153	21	0.140	1.92(0.74-4.97)	0.18
Liver and intrahepatic bile duct	3766	0.159	1350	0.172	0.90(0.83-0.97)	0.01	832	0.180	241	0.160	1.06(0.90-1.26)	0.49	78	0.176	18	0.101	2.42(1.30-4.50)	0.01	802	0.163	285	0.144	1.14(0.97-1.35)	0.11
Gallbladder cancer	563	0.389	1292	0.394	0.80(0.69-0.93)	<0.01	122	0.466	259	0.431	1.12(0.71-1.46)	0.92	11	0.407	22	0.349	0.46(0.10-2.04	0.31	77	0.352	168	0.393	0.80(0.53-1.22)	0.31
Other biliary	797	0.219	776	0.269	0.81(0.71-0.92)	<0.01	104	0.291	113	0.273	0.91(0.64-1.31)	0.63	8	0.205	5	0.147	2.30(0.26-20.83)	0.46	127	0.190	117	0.228	0.77(0.56-1.07)	0.12
Pancreas	13749	0.508	11977	0.475	1.12(1.08-1.17)	<0.01	2099	0.550	2155	0.490	1.25(1.13-1.38)	<0.01	129	0.581	95	0.505	1.41(0.87-2.29)	0.16	1261	0.496	1236	0.449	1.17(1.04-1.32)	0.01
Retroperitoneum	154	0.211	91	0.135	1.62(1.18-2.22)	<0.01	18	0.217	27	0.214	0.67(0.28-1.62)	0.38	0	0.000	0	0.000	-	-	26	0.329	17	0.167	3.12(1.22-7.98)	0.02
Peritoneum, Omentum and Mesentery	28	0.304	782	0.363	1.06(0.59-1.90)	0.85	3	0.333	81	0.424	1.24(0.14-10.86)	0.85	1	0.250	6	0.429	-	-	2	0.286	84	0.410	0.44(0.03-6.41)	0.55
Other Digestive Organs	10	0.189	13	0.277	0.61(0.15-2.48)	0.49	1	0.083	0	0.000	-	-	1	0.500	1	0.500	-	-	0	0.000	2	0.400	-	-
**Respiratory system**	57364	0.466	50069	0.462	1.06(1.04-1.08)	<0.01	9408	0.494	7303	0.492	1.05(1.00-1.10)	0.047	406	0.469	363	0.478	-	-	5911	0.495	4631	0.504	1.15(0.61-2.16)	0.67
Nose, Nasal Cavity and Middle Ear	73	0.056	42	0.052	1.22(0.80-1.85)	0.36	6	0.038	6	0.061	0.71(0.18-2.85)	0.63	0	0.000	0	0.000	-	-	11	0.074	6	0.068	1.82(0.50-6.60)	0.36
Larynx	344	0.034	80	0.033	1.22(0.94-1.58)	0.14	101	0.052	30	0.057	1.07(0.69-1.67)	0.76	5	0.068	0	0.000	-	-	17	0.036	1	0.014	2.58(0.27-24.18)	0.41
Lung and bronchus	56837	0.511	49929	0.476	1.06(1.04-1.08)	<0.01	9292	0.550	7261	0.511	1.05(1.00-1.10)	0.06	400	0.513	362	0.491	0.97(0.77-1.23)	0.80	5721	0.555	4490	0.548	0.82(0.77-0.88)	<0.01
Trachea, Mediastinum and other respiratory organs	110	0.284	18	0.205	1.35(0.70-2.59)	0.37	9	0.220	6	0.300	0.41(0.07-2.44)	0.33	1	0.250	1	0.250	-	-	14	0.304	3	0.214	3.59(0.40-32.16)	0.25
**Bones and joints**	411	0.187	238	0.146	1.22(1.01-1.47)	0.04	49	0.198	36	0.165	1.30(0.74-2.27)	0.36	3	0.125	3	0.158	0.76(0.14-4.29)	0.75	27	0.132	27	0.179	0.67(0.36-1.26)	0.22
**Soft tissue including heart**	1100	0.146	840	0.149	1.00(0.90-1.11)	1.00	191	0.195	161	0.159	1.33(1.03-1.72)	0.03	13	0.167	15	0.250	0.40(0.14-1.18)	0.09	121	0.161	104	0.158	0.99(0.72-1.37)	0.95
**Skin excluding Basal and Squamous Cell**	2988	0.048	1264	0.027	1.76(1.50-2.07)	<0.01	34	0.103	41	0.101	1.47(0.40-5.40)	0.56	8	0.050	6	0.042	-	-	44	0.086	35	0.074	1.10(0.60-2.01)	0.76
Melanoma of the skin	2807	0.047	1206	0.027	1.69(1.57-1.83)	<0.01	28	0.128	35	0.124	1.02(0.53-1.95)	0.96	8	0.056	5	0.039	1.61(0.40-6.43)	0.50	37	0.099	31	0.085	1.10(0.59-2.02)	0.77
Other Non-epithelia skin	181	0.058	58	0.028	2.08(1.50-2.87)	<0.01	6	0.053	6	0.049	4.92(0.47-51.70)	0.18	0	0.000	1	0.067	-	-	7	0.051	4	0.037	1.05(0.05-21.53)	0.98

**Urinary System**	9203	0.090	4338	0.098	1.00(0.84-1.21)	0.97	1067	0.117	617	0.106	1.08(0.81-1.43)	0.62	123	0.164	55	0.129	-	-	667	0.108	315	0.110	1.18(0.99-1.42)	0.07
Urinary Bladder	2349	0.039	936	0.049	0.96(0.88-1.05)	0.35	225	0.066	172	0.093	0.92(0.72-1.17)	0.48	14	0.056	9	0.103	0.46(0.09-2.31)	0.35	137	0.044	50	0.046	1.19(0.81-1.75)	0.37
Kidney and Renal Pelvis	6696	0.162	3281	0.137	1.18(1.11-1.25)	<0.01	832	0.148	430	0.111	1.30(1.11-1.52)	<0.01	109	0.221	45	0.134	1.44(0.87-2.38)	0.16	513	0.175	252	0.154	1.16(0.94-1.43)	0.18
Ureter	131	0.128	105	0.155	0.79(0.56-1.11)	0.17	5	0.147	5	0.135	1.10(0.29-4.20)	0.89	0	0.000	0	0.000	-	-	12	0.111	12	0.117	1.25(0.40-3.90)	0.70
Other Urinary Organs	27	0.089	16	0.105	0.87(0.39-1.91)	0.72	5	0.088	10	0.133	0.63(0.20-1.94)	0.42	0	0.000	1	1.000	-	-	5	0.263	1	0.056	6.07(0.63-58.21)	0.12
**Eye and Orbit**	38	0.018	29	0.017	1.14(0.68-1.92)	0.62	1	0.015	0	0.000	-	-	0	0.000	0	0.000	-	-	0	0.000	2	0.029	-	-
**Brain and other Nervous System**	13	0.065	8	0.050	0.85(0.24-3.00)	0.80	0	0.000	2	0.154	-	-	0	0.000	0	0.000	-	-	0	0.000	0	0.000	-	-
Brain	13	0.065	8	0.050	0.85(0.24-3.00)	0.80	0	0.000	2	0.154	-	-	0	0.000	0	0.000	-	-	0	0.000	0	0.000	-	-
**Endocrine System**	709	0.046	817	0.017	1.29(0.52-3.21)	0.59	57	0.057	150	0.032	1.00(0.48-2.10)	1.00	5	0.043	10	0.023	2.39(0.49-11.74)	0.28	99	0.052	160	0.023	1.64(1.20-2.23)	<0.01
Thyroid	632	0.041	682	0.014	2.01(1.77-2.28)	<0.01	49	0.050	136	0.029	1.13(0.76-1.68)	0.54	4	0.035	10	0.023	2.39(0.49-11.74)	0.28	90	0.048	149	0.022	1.67(1.23-2.29)	<0.01
Other Endocrine including Thymus	77	0.348	135	0.404	0.79(0.52-1.20)	0.27	8	0.296	14	0.333	0.31(0.03-3.08)	0.32	1	0.500	0	0.000	-	-	9	0.321	11	0.379	0.61(0.07-5.20)	0.65
**Lymphoma**	141	0.052	115	0.054	0.87(0.66-1.15)	0.34	20	0.052	23	0.059	0.73(0.34-1.56)	0.42	0	0.000	0	0.000	-	-	10	0.043	6	0.028	2.94(0.65-13.37)	0.16
Hodgkin Lymphoma	7	0.111	6	0.128	2.53(0.22-28.72)	0.46	0	0.000	0	0.000	-	-	0	0.000	0	0.000	-	-	0	0.000	0	0.000	-	-
Non-Hodgkin Lymphoma	134	0.051	109	0.052	0.86(0.65-1.14)	0.30	20	0.053	23	0.061	0.73(0.34-1.56)	0.42	0	0.000	0	0.000	-	-	10	0.043	6	0.028	2.94(0.65-13.37)	0.16
**Myeloma**	33	0.087	16	0.160	0.86(0.36-2.04)	0.73	7	0.065	6	0.154	0.38(0.12-1.21)	0.10	1	0.333	0	0.000	-	-	4	0.160	0	0.000	-	-
**Leukemia**	62	0.075	39	0.133	0.78(0.48-1.26)	0.31	8	0.086	12	0.333	0.33(0.05-2.15)	0.25	0	0.000	0	0.000	-	-	5	0.135	2	0.105	1.33(0.23-7.64)	0.75
**Mesothelioma**	613	0.217	222	0.240	0.85(0.70-1.02)	0.09	48	0.310	21	0.300	0.89(0.40-1.99)	0.78	3	0.176	3	0.429	-	-	29	0.252	13	0.295	0.67(0.25-1.79)	0.43
**Miscellaneous**	54	0.106	40	0.092	1.26(0.78-2.04)	0.35	14	0.233	13	0.165	1.54(0.52-4.58)	0.44	1	0.500	1	0.167	-	-	4	0.143	3	0.094	1.61(0.33-7.88)	0.56
**Total**	122982	0.236	94711	0.258	1.07(1.05-1.09)	<0.01	19630	0.312	15686	0.289	1.08(1.04-1.12)	<0.01	1177	0.269	859	0.239	1.16(1.00-1.33)	0.045	12669	0.266	9884	0.247	1.13(1.04-1.23)	0.01

[a] Adjusted for all confounders: race, marital status, income, insurance, differentiated grade, T stage, and N stage.

Table S3. The prognosis of metastasis and the effect of male sex on it across different non-sex-specific cancer types among different age groups.

Cancer Site	Age<65				Age≥65			
	Median Survival (months)		HR (95%CI) [a]	*P*-value	Median Survival (months)		HR (95%CI) [a]	*P*-value
	Male (Median ±SE)	Female (Median ±SE)			Male (Median ±SE)	Female (Median ±SE)		
Oral Cavity and Pharynx	11.61 ±1.25	11.88 ±0.42	1.08(0.71-1.66)	0.72	7.70 ±0.86	5.45 ±0.68	0.74(0.36-0.90)	<0.01
Lip	13.00±9.17	11.00±0.00	0.54(0.06-1.52)	0.60	11.00±5.66	5.00±1.55	0.34(0.08-1.52)	0.16
Tongue	11.00±0.95	9.00±1.84	1.00(0.66-1.52)	0.98	13.00±5.66	5.00±0.97	0.59(0.38-0.92)	0.02
Salivary Gland	15.00±1.98	22.00±4.88	1.01(0.64-1.59)	0.98	9.00±1.12	10.00±1.61	0.66(0.44-0.99)	0.05
Floor of Mouth	8.00±1.52	15.00±1.06	4.89(1.17-20.41)	0.03	6.00±3.86	5.00±0.95	0.87(0.37-2.03)	0.74
Gum and other mouth	8.00±1.23	12.00±3.29	1.00(0.51-1.98)	1.00	7.00±1.67	4.00±0.78	0.83(0.45-.155)	0.56
Nasopharynx	19.00 ±1.38	26.00 ±7.62	1.87(1.20-2.90)	0.01	10.00±1.22	23.00±10.53	1.23(0.50-3.00)	0.65
Tonsil	16.00±2.58	12.00±1.96	0.64(0.39-1.04)	0.07	8.00±1.56	11.00±3.46	1.08(0.61-1.91)	0.79
Oropharynx	9.00±0.85	3.00±1.71	0.29(0.13-0.64)	<0.01	6.00±2.62	4.00±0.97	0.75(0.33-1.70)	0.49
Hypopharynx	9.00±0.78	8.00±3.93	2.83(1.23-6.51)	0.01	5.00±0.85	5.00±1.58	0.67(0.35-1.30)	0.24
Digestive system	9.91±1.49	14.34±1.80	1.11(1.07-1.15)	<0.01	5.26 ±0.58	6.09 ±0.52	1.07(1.03-1.12)	<0.01
Esophagus	8.00±1.52	15.00±1.06	1.10(0.99-1.22)	0.08	4.00±0.18	4.00±0.41	1.19(1.08-1.32)	<0.01
Stomatch	6.00±0.18	7.00±0.26	1.04(0.98-1.11)	0.16	4.00±0.14	3.00±0.13	1.04(0.98-1.11)	0.23
Small Intestine	51.00±0.18	-	1.31(1.10-1.56)	<0.01	11.00±1.43	13.00±1.71	1.21(1.05-1.40)	0.01
Colon cancer	17.00±0.32	20.00±0.37	1.11(1.06-1.15)	<0.01	7.00±0.23	5.00±0.16	0.98(0.94-1.02)	0.27
Rectum and Rectosigmoid Junction	21.00±0.44	22.00±0.71	1.07(1.01-1.15)	0.03	11.00±0.44	9.00±0.46	1.00(0.93-1.08)	1.00
Anus, Anal Canal and Anorectum	13.00±0.44	23.00±2.74	1.61(1.22-2.14)	<0.01	12.00±2.16	15.00±1.78	1.31(0.92-1.88)	0.13
Liver and intrahepatic bile duct	2.00±0.09	4.00±0.34	1.17(1.07-1.28)	<0.01	2.00±0.11	3.00±0.17	1.12(1.03-1.22)	0.01
Gallbladder cancer	4.00±0.51	6.00±0.30	0.96(0.82-1.13)	0.65	2.00±0.24	3.00±0.16	1.09(0.96-1.24)	0.19
Other biliary	5.00±0.44	7.00±0.67	1.13(0.96-1.34)	0.15	3.00±0.26	3.00±0.28	1.07(0.94-1.22)	0.30

**Pancreas**	**4.00±0.10**	**5.00±0.14**	**1.11(1.07-1.15)**	**<0.01**	**2.00±0.05**	**2.00±0.04**	**1.06(1.02-1.09)**	**<0.01**
**Retroperitoneum**	**21.00±2.55**	**34.00±9.34**	**1.07(0.69-1.67)**	**0.76**	**5.00±1.42**	**8.00±1.44**	**1.45(0.82-2.56)**	**0.20**
**Peritoneum, Omentum and Mesentery**	**26.00±14.89**	**31.00±2.13**	**2.24(1.11-4.52)**	**0.03**	**11.00±3.89**	**20.00±1.36**	**1.42(0.60-3.34)**	**0.43**
**Other Digestive Organs**	**4.00±10.72**	**23.00±11.64**	**0.94(0.25-3.52)**	**0.92**	**7.00±5.39**	**6.00±13.44**	**1.94(0.51-7.40)**	**0.33**
**Respiratory system**	**8.80±1.64**	**10.64±1.70**	**1.20(1.18-1.22)**	**<0.01**	**4.62±1.08**	**5.70±2.53**	**1.39(0.87-2.21)**	**0.17**
**Nose, Nasal Cavity and Middle Ear**	**12.00±2.42**	**15.00±3.40**	**1.13(0.45-2.83)**	**0.79**	**8.00±0.99**	**5.00±3.90**	**0.78(0.33-1.86)**	**0.58**
**Larynx**	**8.00±0.70**	**11.00±3.13**	**1.06(0.70-1.59)**	**0.79**	**6.00±0.67**	**10.00±2.42**	**2.24(1.52-3.28)**	**<0.01**
**Lung and bronchus**	**5.00±0.06**	**8.00±0.09**	**1.20(1.18-1.23)**	**<0.01**	**3.00±0.03**	**3.00±0.04**	**1.17(1.15-1.19)**	**<0.01**
**Trachea, Mediastinum and other respiratory organs**	**19.00±4.48**	**12.00±2.05**	**0.76(0.35-1.66)**	**0.49**	**2.00±0.69**	**2.00±0.00**	**1.64(0.43-6.22)**	**0.47**
**Bones and joints**	**23.00±2.62**	**23.00±3.78**	**0.98(0.78-1.23)**	**0.84**	**5.00±0.71**	**5.00±1.35**	**1.67(0.95-2.95)**	**0.08**
**Soft tissue including heart**	**15.00±0.84**	**15.00±1.00**	**1.17(1.03-1.32)**	**0.02**	**4.00±0.38**	**4.00±0.41**	**0.96(0.82-1.14)**	**0.67**
**Skin excluding Basal and Squamous Cell**	**10.06±0.52**	**13.05±1.10**	**1.13(1.00-1.28)**	**0.053**	**8.35±1.49**	**8.00±0.54**	**1.07(0.95-1.20)**	**0.29**
**Melanoma of the skin**	**10.00±0.52**	**13.00±1.10**	**1.13(1.00-1.28)**	**0.06**	**7.00±0.45**	**8.00±0.73**	**1.07(0.95-1.21)**	**0.28**
**Other Non-epithlia skin**	**13.00±3.64**	**18.00±11.07**	**1.02(0.23-4.46)**	**0.98**	**10.00±1.06**	**8.00±0.80**	**1.00(0.62-1.64)**	**0.98**
**Urinary System**	**8.62±1.27**	**7.79±1.12**	**0.97(0.78-1.20)**	**0.76**	**4.57±0.42**	**3.94±0.46**	**0.98(0.85-1.12)**	**0.72**
**Urinary Bladder**	**7.00±0.37**	**6.00±0.60**	**0.90(0.77-1.04)**	**0.14**	**4.00±0.20**	**3.00±0.23**	**0.91(0.82-1.01)**	**0.08**
**Kidney and Renal Pelvis**	**10.00±0.31**	**9.00±0.42**	**0.99(0.92-1.06)**	**0.67**	**5.00±0.21**	**4.00±0.18**	**0.98(0.92-1.04)**	**0.46**
**Ureter**	**9.00±1.80**	**12.00±3.39**	**3.48(1.23-9.84)**	**0.02**	**5.00±1.39**	**5.00±0.59**	**0.95(0.66-1.37)**	**0.79**
**Other Urinary Organs**	**18.00±0.00**	**7.00±1.42**	**0.41(0.14-1.21)**	**0.11**	**5.00±1.40**	**6.00±2.61**	**3.33(1.35-8.25)**	**0.01**
**Eye and Orbit**	**13.00±12.01**	**35.00±9.75**	**0.88(0.10-7.63)**	**0.91**	**5.00±1.18**	**19.00±7.32**	**2.35(0.94-5.89)**	**0.07**
**Brain and other Nervous System**	**-**	**43.00±32.08**	**0.88(0.12-6.45)**	**0.90**	**18.00±11.31**	**12.00±4.49**	**0.61(0.18-2.06)**	**0.43**
**Brain**	**-**	**43.00±32.08**	**0.88(0.12-6.45)**	**0.90**	**18.00±11.31**	**12.00±4.49**	**0.61(0.18-2.06)**	**0.43**
**Endocrine System**	**7.00±1.72**	**7.00±0.96**	**1.27(1.03-1.56)**	**0.03**	**6.32±2.49**	**4.35±2.50**	**0.97(0.80-1.18)**	**0.75**
**Thyroid**	**-**	**-**	**1.33(1.05-1.69)**	**0.02**	**9.00±1.67**	**7.00±1.52**	**0.95(0.78-1.16)**	**0.61**

**Other Endocrine including Thymus**	**7.00±1.72**	**7.00±0.96**	**1.07(0.69-1.65)**	**0.76**	**4.00±0.98**	**2.00±0.87**	**1.50(0.59-1.89)**	**0.40**
**Lymphoma**	**-**	**-**	**0.88(0.29-2.69)**	**0.83**	**16.33±15.50**	**24.00±4.70**	**1.19(0.75-1.89)**	**0.74**
**Hodgkin Lymphoma**	**-**	**-**	**0.61(0.04-9.93)**	**0.73**	**1.00±1.50**	**20.00±0.00**	**0.61(0.04-9.61)**	**0.73**
**Non-Hodgkin Lymphoma**	**-**	**-**	**0.95(0.28-3.20)**	**0.93**	**32.00±4.30**	**24.00±4.70**	**1.19(0.75-1.89)**	**0.47**
**Myeloma**	**10.00±5.80**	**6.00±1.31**	**0.67(0.24-1.88)**	**0.45**	**16.00±4.10**	**12.00±9.99**	**0.28(0.06-1.23)**	**0.09**
**Leukemia**	**24.00±7.54**	**31.00±17.60**	**0.75(0.16-3.62)**	**0.72**	**11.00±2.52**	**14.00±2.74**	**4.17(1.54-11.28)**	**0.01**
**Mesothelioma**	**8.00±1.86**	**11.00±1.81**	**0.92(0.65-1.32)**	**0.66**	**4.00±0.39**	**8.00±0.94**	**1.42(1.12-1.79)**	**<0.01**
**Miscellaneous**	**12.00±5.51**	**10.00±3.31**	**1.38(0.43-4.44)**	**0.59**	**15.00±6.73**	**21.00±10.32**	**0.93(0.33-2.63)**	**0.89**
**Total**	**10.94±0.35**	**10.66±0.42**	**1.18(1.16-1.20)**	**<0.01**	**4.71±0.19**	**5.47±0.21**	**1.06(1.02-1.10)**	**<0.01**

[a] Adjusted for all confounders: race, marital status, income, insurance, differentiated tumor grade, T stage, N stage, number of metastatic sites, surgery on the primary site, receiving radiation therapy, and receiving chemotherapy.

Table S4. The prognosis of metastasis and the effect of male sex on it across different non-sex-specific cancer types among different race groups.

Cancer Site	White				Black				Asian			
	Median Survival (months)		HR (95%CI) [a]	*P*-value	Median Survival (months)		HR (95%CI) [a]	*P*-value	Median Survival (months)		HR (95%CI) [a]	*P*-value
	Male (Median±SE)	Female (Median±SE)			Male (Median±SE)	Female (Median±SE)			Male (Median±SE)	Female (Median±SE)		
**Oral Cavity and Pharynx**	10.61±1.12	8.45±1.42	0.84(0.67-1.06)	0.14	8.16±0.77	4.92±0.84	0.87(0.40-1.93)	0.74	8.26±2.06	5.12±4.49	0.81(0.42-1.56)	0.53
Lip	15.00±2.34	5.00±5.50	0.27(0.07-1.00)	0.0.54	-	-	-	-	-	-	-	-
Tongue	13.00±0.84	7.00±1.58	0.75(0.54-1.03)	0.08	7.00±1.11	5.00±4.33	1.33(0.51-0.49)	0.56	8.00±1.87	1.00±0.75	0.20(0.04-0.95)	0.04
Salivary Gland	12.00±1.26	14.00±1.67	0.80(0.59-1.10)	0.17	10.00±1.70	11.00±8.57	0.29(0.02-4.85)	0.39	28.00±16.07	24.00±9.11	0.55(0.18-1.68)	0.29
Floor of Mouth	8.00±1.84	7.00±2.04	0.63(0.19-2.12)	0.46	4.00±2.25	-	4.91(1.04-23.27)	0.045	5.00±0.82	-	-	-
Gum and other mouth	6.00±0.92	5.00±1.75	0.80(0.51-1.26)	0.34	-	-	0.88(0.40-1.94)	0.75	7.00±2.00	2.00±4.71	1.13(0.27-4.79)	0.87
Nasopharynx	16.00±2.84	18.00±6.01	1.38(0.75-2.51)	0.30	12.00±2.62	19.00±6.71	3.07(1.08-8.74)	0.04	17.00±1.51	51.00±0.00	1.33(0.70-2.53)	0.38
Tonsil	13.00±1.63	14.00±2.34	0.94(0.64-1.39)	0.77	9.00±2.27	5.00±0.65	0.27(0.03-2.56)	0.25	7.00±2.78	-	-	-
Oropharynx	8.00±0.97	4.00±1.76	0.47(0.25-0.91)	0.03	9.00±2.04	3.00±1.79	0.25(0.03-2.19)	0.21	3.00±2.62	3.00±0.00	1.15(0.12-10.77)	0.90
Hypopharynx	8.00±0.99	7.00±1.60	1.50(0.88-2.58)	0.14	8.00±1.29	5.00±1.65	0.22(0.07-0.68)	0.01	8.00±4.06	-	-	-
**Digestive system**	8.60±0.88	10.81±1.09	1.09(1.05-1.12)	<0.01	7.32±0.90	7.68±1.10	1.07(1.02-1.11)	<0.01	8.37±1.05	7.86±1.08	1.08(1.02-1.15)	0.02
Esophagus	5.00±0.13	5.00±0.87	1.13(1.04-1.23)	<0.01	4.00±0.29	4.00±0.51	1.32(1.08-1.61)	0.01	6.00±0.55	8.00±2.26	1.33(0.90-1.96)	0.16
Stomach	5.00±0.14	5.00±0.20	1.05(1.00-1.11)	0.06	4.00±0.32	5.00±0.35	1.07(0.95-1.19)	0.27	5.00±0.36	6.00±0.40	0.94(0.84-1.06)	0.33
Small Intestine	29.00±2.70	34.00±3.65	1.21(1.07-1.37)	<0.01	13.00±1.90	25.00±4.53	1.18(0.91-1.54)	0.22	13.00±3.72	22.00±8.55	1.67(0.92-3.01)	0.09
Colon cancer	12.00±0.22	11.00±0.25	1.03(1.00-1.07)	0.04	12.00±0.50	11.00±0.48	1.04(0.98-1.11)	0.23	16.00±0.81	14.00±0.84	1.07(0.96-1.18)	0.23
Rectum and Rectosigmoid Junction	18.00±0.40	17.00±0.51	1.05(0.99-1.11)	0.10	15.00±0.86	12.00±1.07	1.03(0.90-1.18)	0.65	20.00±1.19	17.00±1.77	1.07(0.91-1.26)	0.42
Anus, Anal Canal and Anorectum	13.00±1.51	21.00±1.84	1.52(1.21-1.91)	<0.01	13.00±2.97	21.00±4.90	2.18(0.86-5.55)	0.10	7.00±2.78	13.00±7.47	1.00(0.43-2.32)	1.00
Liver and intrahepatic bile duct	3.00±0.09	3.00±0.23	1.15(1.07-1.24)	<0.01	2.00±0.17	2.00±0.36	1.08(0.91-1.27)	0.39	2.00±0.15	3.00±0.40	1.19(1.02-1.40)	0.03
Gallbladder cancer	3.00±0.25	4.00±0.22	1.08(0.96-1.21)	0.18	4.00±0.44	4.00±0.55	0.87(0.66-1.15)	0.32	4.00±0.73	4.00±0.52	0.97(0.68-1.38)	0.87
Other biliary	4.00±0.29	4.00±0.32	1.06(0.94-1.18)	0.35	3.00±0.72	4.00±0.93	1.13(0.81-1.58)	0.46	5.00±0.82	7.00±0.75	1.20（0.86-1.67）	0.28
Pancreas	3.00±0.05	3.00±0.05	1.07(1.04-1.10)	<0.01	2.00±0.12	2.00±0.12	1.07(1.00-1.15)	0.04	3.00±0.18	3.00±0.14	1.11(1.01-1.21)	0.03
Retroperitoneum	13.00±3.33	15.00±3.19	0.94(0.64-1.37)	0.73	16.00±1.85	45.00±24.24	1.53(0.66-3.55)	0.32	20.00±3.03	18.00±7.84	2.24(0.52-9.59)	0.28
Peritoneum, Omentum and Mesentery	11.00±4.18	23.00±1.20	1.49(0.88-2.53)	0.14	20.00±16.33	21.00±3.31	1.91(0.30-12.24)	0.49	-	-	-	-
Other Digestive Organs	4.00±7.73	23.00±9.98	1.53(0.55-4.28)	0.42	-	-	-	-	-	6.00±0.00	-	-
**Respiratory system**	7.78±1.90	-	1.22(1.02-1.47)	0.03	6.34±1.42	6.80±1.90	1.27(0.87-1.84)	0.22	7.60±1.33	6.83±3.91	1.30(1.24-1.36)	<0.01
Nose, Nasal Cavity and Middle Ear	10.00±1.69	11.00±3.54	0.87(0.52-1.45)	0.60	9.00±1.67	19.00±8.03	16.18(0.87-299.60)	0.06	12.00±2.84	2.00±3.67	0.81(0.24-2.70)	0.73
Larynx	7.00±0.67	11.00±1.96	1.56(1.14-2.14)	0.01	7.00±0.64	8.00±1.96	1.56(0.81-3.00)	0.18	8.00±1.34	10.00±0.00	0.57(0.06-5.10)	0.62
Lung and bronchus	3.00±0.04	4.00±0.05	1.18(1.16-1.19)	<0.01	4.00±0.09	5.00±0.13	1.14(1.10-1.18)	<0.01	6.00±0.18	10.00±0.35	1.30(1.34-1.36)	<0.01
Trachea, Mediastinum and other respiratory organs	13.00±2.24	33.00±17.15	1.41(0.59-3.37)	0.44	14.00±0.00	2.00±0.00	0.95(0.17-5.19)	0.95	10.00±0.00	12.00±0.00	1.00(0.14-6.93)	1.00
**Bones and joints**	19.00±1.65	16.00±2.89	1.10(0.88-1.39)	0.40	15.00±3.81	18.00±3.43	1.41(0.61-3.24)	0.42	28.00±10.23	20.00±3.06	0.40(0.11-1.40)	0.15
**Soft tissue including heart**	11.00±0.69	12.00±0.92	1.03(0.92-1.15)	0.66	10.00±1.32	11.00±1.34	1.22(0.92-1.62)	0.17	8.00±1.62	11.00±2.22	1.28(0.88-1.86)	0.20
**Skin excluding Basal and Squamous Cell**	8.80±0.98	9.13±0.99	1.10(1.01-1.20)	0.02	7.64±1.81	11.00±1.80	2.51(1.08-5.80)	0.03	11.81±6.90	8.14±2.00	1.13(0.41-3.10)	0.81
Melanoma of the skin	8.00±0.36	10.00±0.67	1.11(1.02-1.21)	0.02	8.00±1.88	11.00±2.23	3.22(1.14-9.10)	0.03	6.00±2.34	6.00±1.37	0.82(0.36-1.89)	0.64
Other Non-epithlia skin	10.00±0.97	8.00±0.98	0.94(0.61-1.44)	0.78	3.00±6.74	11.00±3.06	1.57(0.38-6.51)	0.53	20.00±6.24	10.00±0.87	2.58(0.47-14.22)	0.28
**Urinary System**	6.28±1.05	5.17±0.77	0.94(0.89-1.00)	0.048	4.80±1.13	5.23±0.74	1.04(0.92-1.17)	0.58	7.35±0.55	5.53±0.60	0.99(0.83-1.18)	0.92

Urinary Bladder	5.00±0.21	4.00±0.27	0.90(0.82-0.98)	0.02	5.00±6.17	4.00±0.52	0.88(0.69-1.12)	0.31	7.00±0.76	5.00±0.87	1.12(0.69-1.83)	0.65
Kidney and Renal Pelvis	8.00±0.21	6.00±0.22	0.95(0.90-1.00)	0.06	6.00±0.41	6.00±0.63	1.09(0.95-1.26)	0.21	8.00±0.87	6.00±0.84	0.96(0.79-1.16)	0.65
Ureter	6.00±1.09	5.00±0.74	1.22(0.88-1.70)	0.23	6.00±1.10	7.00±3.29	1.26(0.23-6.82)	0.79	6.00±2.44	6.00±4.04	1.62(0.58-4.51)	0.36
Other Urinary Organs	6.00±0.97	10.00±4.03	1.37(0.17-10.83)	0.77	2.00±1.10	6.00±1.41	1.14(0.36-3.63)	0.83	7.00±3.29	3.00±0.00	0.41(0.03-6.62)	0.53
Eye and Orbit	10.00±1.32	21.00±5.99	1.44(0.46-4.54)	0.53	-	-	-	-	-	6.00±0.00	-	-
Brain and other Nervous System	25.00±12.98	12.00±7.78	0.73(0.25-2.12)	0.56	-	-	-	-	-	-	-	-
Brain	25.00±12.98	12.00±7.78	0.73(0.25-2.12)	0.56	-	-	-	-	-	-	-	-
Endocrine System	13.10±7.49	13.00±8.94	1.04(0.89-1.22)	0.61	2.00±1.29	10.51 ±11.11	1.44(0.87-2.37)	0.16	13.51±5.98	22.85±17.30	1.26(0.73-2.18)	0.41
Thyroid	21.00±3.67	23.00±6.07	1.05(0.88-1.24)	0.61	31.00±0.00	25.00±12.09	1.56(0.89-2.74)	0.12	20.00±4.30	43.00±14.22	1.43(0.90-2.25)	0.13
Other Endocrine including Thymus	6.00±1.25	5.00±0.85	1.00(0.68-1.47)	0.99	2.00±1.29	2.00±2.95	1.05(0.35-3.15)	0.93	8.00±2.61	8.00±4.06	0.68(0.19-2.40)	0.55
Lymphoma	20.19±17.98	44.00±10.40	1.32(0.86-2.03)	0.21	-	-	0.58(0.21-1.56)	0.28	9.00±5.53	-	-	-
Hodgkin Lymphoma	3.00±1.31	-	2.79(0.47-16.38)	0.26	-	-	-		-	-	-	-
Non-Hodgkin Lymphoma	39.00±7.75	44.00±10.40	1.26(0.81-1.96)	0.31	50.00±0.00	57.00±0.00	0.58(0.21-1.56)	0.28	9.00±5.53	-	1.61(0.32-8.11)	0.56
Myeloma	16.00±5.82	12.00±8.77	0.41(0.09-1.89)	0.26	16.00±1.31	5.00±2.04	0.31(0.08-1.17	0.08	10.00±8.00	-	-	--
Leukemia	15.00±4.28	18.00±1.94	1.29(0.60-2.76)	0.51	7.00±4.24	45.00±19.08	1.85(0.58-5.91)	0.30	2.00±1.10	20.00±0.00	3.77(0.40-35.47)	0.25
Mesothelioma	5.00±0.48	9.00±0.88	1.29(1.05-1.58)	0.02	2.00±0.68	5.00±3.64	0.90(0.34-2.42)	0.84	4.00±1.30	7.00±1.60	0.02(0.00-0.65)	0.03
Miscellaneous	17.00±7.83	14.00±7.42	0.88(0.44-1.76)	0.72	12.00±3.74	8.00±4.79	0.86(0.34-2.42)	0.72	8.00±.0.00	26.00±4.90	3.53(0.23-55.08)	0.37
Total	8.13 ±0.29	9.36 ±0.38	1.06(1.03-1.09)	<0.01	6.37 ±0.34	6.69 ±0.42	1.07(1.03-1.11)	<0.01	6.73 ±0.38	6.97 ±0.46	1.21(1.16-1.25)	<0.01

[a] Adjusted for all confounders: race, marital status, income, insurance, differentiated tumor grade, T stage, N stage, number of metastatic sites, surgery on the primary site, receiving radiation therapy, and receiving chemotherapy.
